# Tietze Syndrome as a Cause of Chest Pain in the Post-COVID-19 Period

**DOI:** 10.7759/cureus.37360

**Published:** 2023-04-10

**Authors:** Nika Kuridze, Ivditi Okuashvili, Mikheil Tsverava, Eteri Minadze

**Affiliations:** 1 Faculty of Clinical and Translational Medicine, Ivane Javakhishvili Tbilisi State University, Tbilisi, GEO; 2 Rhythmology Department, G. Chapidze Emergency Cardiology Center, Tbilisi, GEO; 3 Outpatient Department, G. Chapidze Emergency Cardiology Center, Tbilisi, GEO; 4 Faculty of Internal Medicine, Ivane Javakhishvili Tbilisi State University, Tbilisi, GEO

**Keywords:** sars-cov-2 virus, post-covid-19 period, covid-19, chest pain, tietze syndrome

## Abstract

Tietze syndrome is a rare disease. It is mainly characterized by chest pain caused by a unilateral and monoarticular lesion of the second-fifth costal joints. Tietze syndrome is one of the potential complications in the post-COVID-19 period. It is one of the differential diagnoses for non-ischemic chest pain. With early diagnosis and appropriate treatment, this syndrome is easily manageable. The authors present a case of a 38-year-old male who had been diagnosed with Tietze syndrome in the post-coronavirus disease 2019 (COVID-19) period.

## Introduction

Tietze syndrome was first described in 1921 by German surgeon Alexander Tietze. True Tietze syndrome is rare, mainly affecting people under 40. Its etiology is not well known. Presumably, frequent and severe coughing, vomiting, and microtraumas caused by physical exertion all have a part in the development of Tietze syndrome [1]. It’s characterized by chest pain and swelling of the cartilage of one or more upper ribs, specifically where the ribs attach to the sternum. In some cases, pain may cover not only the chest region but the arm and shoulder as well. Tietze syndrome has been associated with viral or bacterial infections, albeit some post-coronavirus disease 2019 (COVID-19) cases have been reported [2].

## Case presentation

A 38-year-old male visited our hospital's outpatient department. The reason for admission was acute chest pain. He had confirmed severe acute respiratory syndrome coronavirus 2 (SARS-CoV-2) by polymerase chain reaction test (PCR test) six weeks ago. According to the patient, he did not receive any prior vaccinations against COVID-19, the course of the disease was mild, and he experienced a subfebrile temperature (max: 37.7 °C) for three days, but no other symptoms emerged. At home, he was self-isolated. The patient had II-grade arterial hypertension, and he was taking captopril irregularly without another medical history. There was no history of inherited disorders, chronic illnesses, or surgical intervention. The patient complained of moderate, persistent pain at the sternum on the left parasternal line, which was exacerbated with deep inhalation. The symptoms mentioned above started five days before the patient's visit to the hospital, approximately six weeks after COVID-19 was diagnosed. During this time, he did not exhibit signs of other illnesses, had no injuries, and the patient himself could not relate the pain to anything else. According to an inspection, the patient's skin surface was swollen at the fourth manubriosternal joint. The skin colour remained unaltered, and palpation of the aforementioned location was painful. At admission, the heart rate was 76 beats per minute, and the blood pressure was 130/80 mmHg. Laboratory tests, such as complete blood count, high-sensitivity cardiac troponin and C-reactive protein, as well as electrocardiogram and echocardiography, revealed no abnormalities.

**Figure 1:**
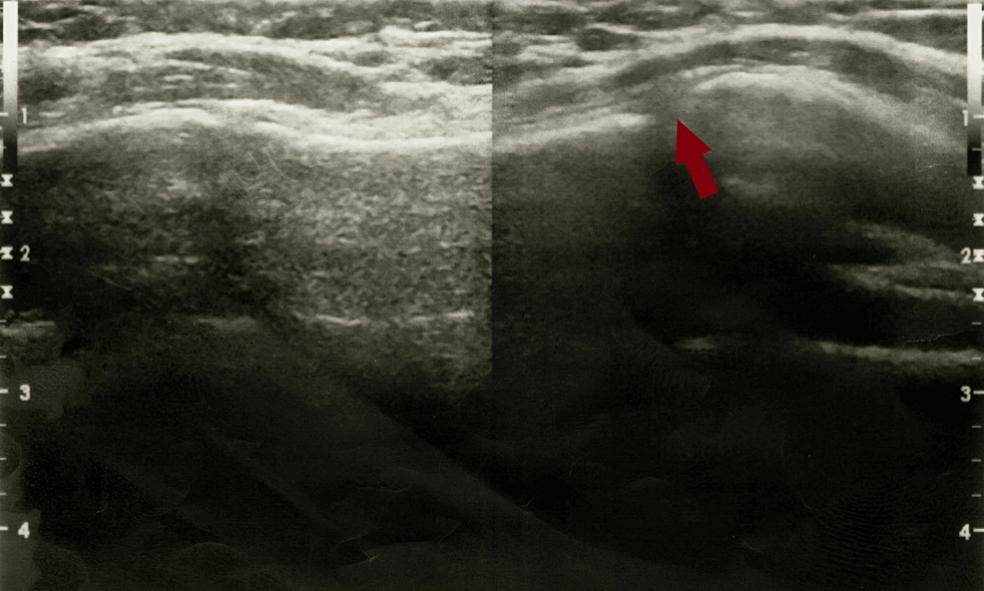
Ultrasound scan image of the sternocostal joints done during the first visit Ultrasound scan image of the sternocostal joints at the first visit shows an oval-shaped hypoechoic focus which is typical for Tietze syndrome.

Considering the patient's complaints, an ultrasound scan of the sternocostal joints was performed using a high-frequency 10 MHz transducer. During the examination, an oval-shaped hypoechoic focus measuring 16mm/8mm/7mm was discovered at the 4th manubriosternal joint, distorting the joint contour (Figure 1). 

Color Flow Doppler imaging revealed no vascularization in the affected joint region. Other sternocostal joints, both right and left, remained unaltered. Based on the clinical and instrumental examination findings, the patient was diagnosed with Tietze syndrome and was prescribed ibuprofen 600 mg three times a day for one week.

**Figure 2:**
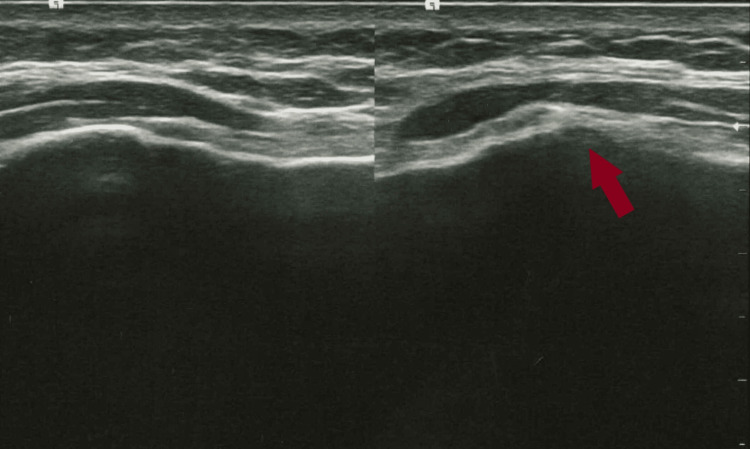
The Image of an ultrasound scan of the sternocostal joints at the follow-up visit

At the follow-up visit, one week following the first consultation, the patient noticed that after 24 hours of taking ibuprofen, the swelling and severity of the pain had diminished. After a week, the swelling and pain had completely subsided, and dynamic ultrasonography of the costochondral joint could no longer identify the previously noted hypoechoic region (Figure 2).

## Discussion

Tietze syndrome is characterized by aseptic costal cartilage damage and painful swelling of the joint area caused by an inflammatory response. Pain is exacerbated by deep inhalation and vigorous chest movement. The targets of Tietze syndrome are the sternocostal, costochondral, and sternoclavicular junctions. In 70-80% of clinical cases, the process is unilateral and monoarticular, primarily affecting the level of the second and third ribs. Tietze syndrome may be accompanied by an increase in body temperature and the appearance of erythema [3,4].

Age less than 40 years, primarily unilateral and monoarticular lesion of the second-fifth costal joints, and inspection findings distinguish Tietze syndrome from other potentially life-threatening disorders characterized by chest pain (acute coronary syndrome, etc.) [5].

In the case of a patient mentioned earlier, the presence of COVID-19 in the health history, unilateral and monoarticular painful swelling of the costal joint, together with relevant ultrasonography alterations, led us to assume Tietze syndrome.

Chest X-ray, ultrasonography, computed tomography, radionuclide scintigraphy, magnetic resonance imaging, and positron emission tomography all play important roles in diagnosing Tietze syndrome [6]. The ultrasound scan obviously indicates inflammatory changes in the sternocostal joints and swelling of the intraarticular tissue, the image is precise enough that the patient typically doesn't need any further diagnostic procedures. During this time, an ultrasound demonstrates an increase in the dimensions of the articular surfaces, cartilage, and surrounding structural components, as well as a reduction in their echogenicity. Color Flow Doppler imaging may occasionally show increased vascularization of the injured joint structures. This is not, however, a defining characteristic of Tietze syndrome.

Tietze syndrome is a self-limiting disease with frequent relapses. The pain might disappear in a few weeks or continue for months. Nonsteroidal anti-inflammatory drugs (NSAIDs) are often used in treatment [7]. Applying warm compresses to the affected region may alleviate pain. Colchicine is sometimes used as an alternative NSAIDs [8]. In severe cases, intra-articular injections of glucocorticoids are employed, and surgical therapy is seldom used [9].

## Conclusions

We believe this is one of the first clinical cases of Tietze syndrome in a post-COVID-19 period. When a patient with chest pain is admitted to the hospital in the post-COVID-19 period, Tietze syndrome should be included among the differential diagnoses. If this syndrome is suspected, its diagnosis does not require huge financial costs and complex invasive interventions. As for its prognosis, under the conditions of proper treatment, it is benign.
